# Radiotherapy concepts for spinal metastases—results from an online survey among radiation oncologists of the German Society for Radiation Oncology

**DOI:** 10.1007/s00066-023-02082-w

**Published:** 2023-06-05

**Authors:** Maria Waltenberger, Marco M. E. Vogel, Denise Bernhardt, Stefan Münch, Sophie Dobiasch, Kristin J. Redmond, Simon S. Lo, Güliz Acker, Michael G. Fehlings, Florian Ringel, Peter Vajkoczy, Bernhard Meyer, Stephanie E. Combs

**Affiliations:** 1 Department of Radiation Oncology, Klinikum rechts der Isar, Technical University of Munich (TUM), Munich, Germany; 2 German Cancer Consortium (DKTK), partner site Munich, Munich, Germany; 3 German Cancer Research Center (DKFZ), Heidelberg, Germany; 4 Institute of Radiation Medicine (IRM), Helmholtz Zentrum, Munich, Germany; 5 Department of Radiation Oncology and Molecular Radiation Sciences, Johns Hopkins University School of Medicine, Baltimore, USA; 6 Department of Radiation Oncology, University of Washington School of Medicine, Seattle, USA; 7 Department of Neurosurgery, Charité Universitätsmedizin Berlin (Corporate Member of Freie Universität Berlin, Humboldt-Universität zu Berlin, and Berlin Institute of Health), Berlin, Germany; 8 Department of Radiation Oncology, Charité Universitätsmedizin Berlin (Corporate Member of Freie Universität Berlin, Humboldt-Universität zu Berlin, and Berlin Institute of Health), Berlin, Germany; 9 Berlin Institute of Health, Berlin, Germany; 10 Division of Neurosurgery, Department of Surgery, University of Toronto, Toronto, Canada; 11 Department of Neurosurgery, University Hospital Mainz, Mainz, Germany; 12 Department of Neurosurgery, Klinikum rechts der Isar, Technical University of Munich (TUM), Munich, Germany

**Keywords:** Spinal metastases, Radiotherapy, Palliation, SBRT, Patterns of care, Online survey

## Abstract

**Purpose:**

Spinal metastases (SM) are a common radiotherapy (RT) indication. There is limited level I data to drive decision making regarding dose regimen (DR) and target volume definition (TVD). We aim to depict the patterns of care for RT of SM among German Society for Radiation Oncology (DEGRO) members.

**Methods:**

An online survey on conventional RT and Stereotactic Body Radiation Therapy (SBRT) for SM, distributed via e‑mail to all DEGRO members, was completed by 80 radiation oncologists between February 24 and April 29, 2022. Participation was voluntary and anonymous.

**Results:**

A variety of DR was frequently used for conventional RT (primary: *n* = 15, adjuvant: *n* = 14). 30 Gy/10 fractions was reported most frequently. TVD in adjuvant RT was heterogenous, with a trend towards larger volumes. SBRT was offered in 65% (primary) and 21% (adjuvant) of participants’ institutions. A variety of DR was reported (primary: *n* = 40, adjuvant: *n* = 27), most commonly 27 Gy/3 fractions and 30 Gy/5 fractions. 59% followed International Consensus Guidelines (ICG) for TVD.

**Conclusion:**

We provide a representative depiction of RT practice for SM among DEGRO members. DR and TVD are heterogeneous. SBRT is not comprehensively practiced, especially in the adjuvant setting. Further research is needed to provide a solid data basis for detailed recommendations.

## Introduction

Spinal metastases are one of the common indications for radiotherapy (RT), given a prevalence of up to 50% [1] in cancer patients. RT of spinal metastases may be performed using conventional RT in palliative intent [2, 3] although ablative treatment with Stereotactic Body Radiation Therapy (SBRT) in both palliative and potentially curative intent is being increasingly utilized [4–6].

The primary goal of conventional palliative RT is pain control. Various fractionation schemes are accepted and recommended, for instance 8 Gy in a single fraction, 20 Gy in 4 fractions and 30 Gy in 10 fractions [5, 7–9]. It is known, however, that RT schemes with higher biologically effective dose lead to improved long term tumor control [10, 11]. This is becoming increasingly important as survival in metastatic cancer patients increases due to modern effective systemic therapies. SBRT is a locally ablative therapy performed in the context of oligometastatic disease in a potentially curative scenario [6, 12] and is also offered for pain control in the palliative setting [5, 13]. As in conventional palliative RT, dose schemes used in SBRT of spinal metastases show a considerable range of variation. Dose regimens used in large prospective studies range from 16 or 20 Gy in a single fraction [6, 14] to 24 Gy in 2 fractions [5], 30 Gy in 3 fractions or 35 Gy in 5 fractions [6].

Regarding target volume definition, a heterogeneity of contouring strategies exists. International Consensus Guidelines (ICG) for target volume delineation are available for primary and adjuvant SBRT [15–17], however, there is no uniform recommendation for palliative RT. This is crucial especially in the adjuvant setting, where surgical instrumentation and surgical tract might or might not be included in the target volume.

There is an ongoing interdisciplinary discussion between spine surgeons and radiation oncologists on the optimal sequence of RT for primary and secondary tumors, especially with respect to the potential impact of including all areas touched during surgery. For spinal metastases, the discussion questions the traditional and well-established concept of including all affected areas, vertebrae above and below the affected region, and the whole instrumentation, when applicable.

The aim of the survey was to provide a representative depiction of the patterns of care for spinal metastases from a radiation oncology perspective among members of the German Society for Radiation Oncology (Deutsche Gesellschaft für Radioonkologie e.V., DEGRO). The study aims to provide an overview of the daily practice in terms of dose prescription and target volume definition, as well as frequency of treatments and RT techniques used.

## Materials and methods

The survey on RT treatment concepts for spinal metastases was developed by a team of radiation oncologists. All questions were specifically developed for the purpose of this investigation, and they were asked in German language. The questions were reviewed by experienced national and international radiation oncologists and a neurosurgeon specializing in radiosurgery, all members of the Spine Tumor Academy (STA).

The questionnaire (see Appendix for the original questionnaire and an English translation) consisted of four thematic sections: Conventional RT in primary (1) and post-operative, adjuvant (2) palliative intention as well as primary (3) and adjuvant (4) SBRT for spinal metastases. Participant-specific information was also obtained. The survey consisted of a total of 31 questions, three of which participant-specific (level of seniority, type of institution, country of practice), 16 related to conventional RT and 12 related to SBRT. Not all questions had to be answered by all participants: Participants from institutions where SBRT is not practiced answered a total of 20 questions, and participants at institutions where spinal SBRT is offered as primary treatment answered a total of 26 questions. The following types of questions were used: Short text, single choice and multiple choice. The questionnaire was available as an online survey at survio.com. Survio is an online platform for survey generation, powered by Survio s.r.o. (Brno, Czech Republik). The platform ensures data safety (ISO/IEC 27001 certificate, 2048-bit SSL security, ISO/IEC 270001 standards, daily backups).

A call for participation was sent via the DEGRO membership mailing list on February 24, 2022. A second call/reminder was emailed on March 15, 2022. A total of 1162 active radiation oncologists, representing the target group of this survey, were reached via this mailing list. The target group consisted of *n* = 217 physicians in residency training and *n* = 945 with completed residency training. Participation was voluntary and anonymous. A total of 80 radiation oncologists completed the survey between February 24 and April 29, 2022, corresponding to a total response rate of approximately 7% and a response rate of approximately 8% among radiation oncologists with completed residency training. Statistical analyses were carried out with SPSS version 27 (IBM, Armonk, NY, USA). Percentages are reported rounded to the nearest whole number.

## Results

**Fig. 1:**
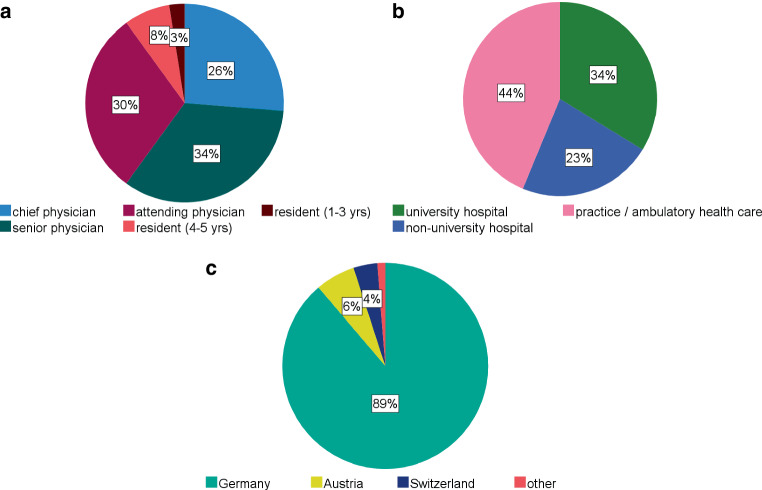
Pie charts of participant-specific results with **a** (upper left) illustrating proportions of level of seniority, **b** (upper right) showing type of institution and **c** (bottom) country of practice of the *n* = 80 participants of the survey

A graphical overview of the results on the participants of the survey can be found in Fig. 1. Most of the 80 participants (*n* = 72, 90%) have completed residency training in radiation oncology. In detail, the level of seniority of the participants was as follows: *N* = 24 (30%) participants were attending physicians (German: Facharzt), *n* = 27 (34%) senior physicians (German: Oberarzt), and *n* = 21 (26%) chief physicians (German: Chefarzt/Leitung einer Einrichtung). Of the *n* = 8 (10%) participants in residency training, *n* = 2 (2%) had 1–3 years and *n* = 6 (8%) had 4–5 years of working experience. Participants’ institutions were about evenly split between hospitals (*n* = 45, 56%) and medical practice or ambulatory health care (German: Medizinisches Versorgungszentrum, MVZ; *n* = 35, 44%). Of the radiation oncologists who practice in hospitals, 60% (*n* = 27) work in a university hospital and 40% (*n* = 18) in a non-university institution. The majority (*n* = 71, 89%) of participants stated that they are based in Germany. Radiation oncologists practicing in Austria (*n* = 5, 6%), Switzerland (*n* = 3, 4%) and Cyprus (*n* = 1, 1%) also participated in the survey.

### Section 1—conventional RT in primary palliative intention

Regarding patient volume, participants stated that the annual number of patients with spinal metastases treated in primary palliative intention at their institution is 1–50 in 36% (*n* = 29), 51–100 in 31% (*n* = 25), 101–300 in 25% (*n* = 20), 301–500 in 1% (*n* = 1), 501–1000 in 5% (*n* = 4) and >1000 in 1% (*n* = 1).

Most participants (*n* = 51, 64%) performed treatment planning for conventional primary palliative RT computed tomography (CT)-based only, while 34% (*n* = 29) used magnetic resonance imaging (MRI) for treatment planning as well. The most used irradiation technique in this setting was Volumetric Modulated Arc Therapy (VMAT), with 66% (*n* = 53) of radiation oncologists applying it frequently, followed by (3D-RT), which was frequently used by 50% (*n* = 40) of the participants. Other techniques that were reported as most frequently used are intensity-modulated RT (IMRT) (26%, *n* = 21), Tomotherapy (5%, *n* = 4) and anterior-posterior posterior-anterior fields (ap-pa) (1%, *n* = 1).

**Fig. 2:**
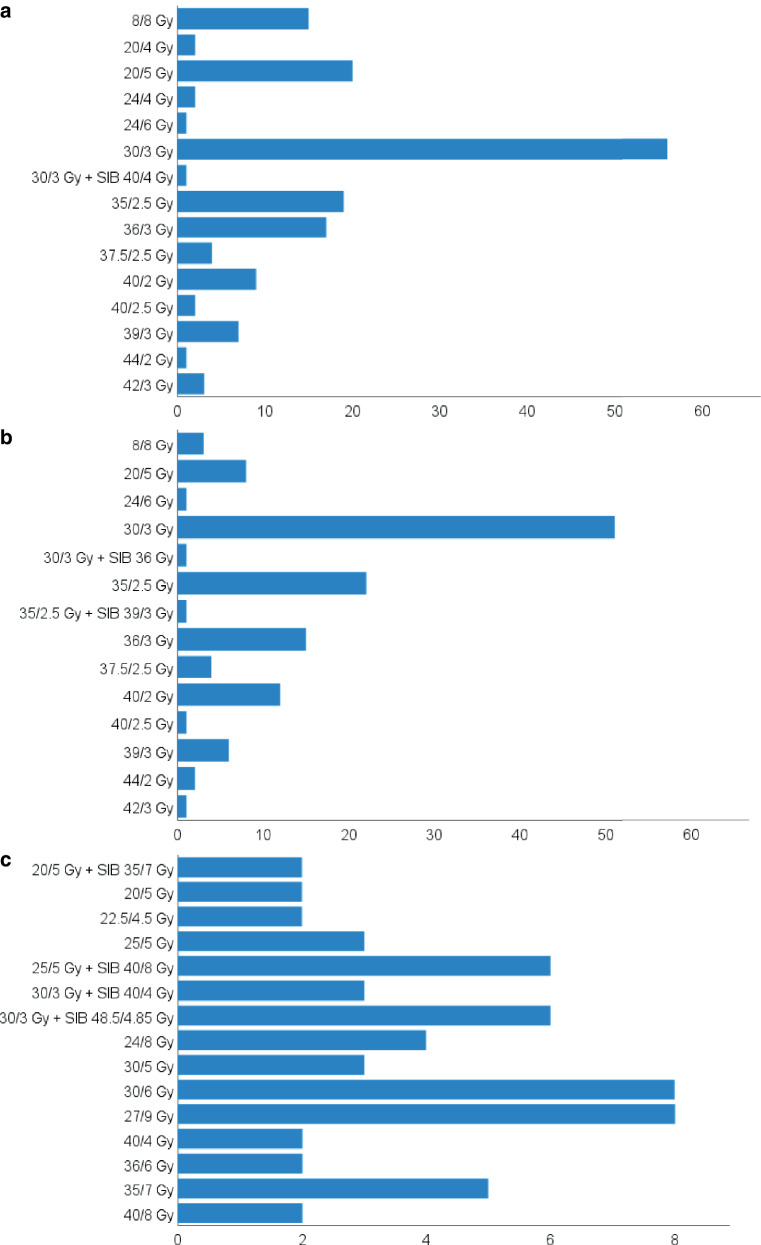
Bar charts for dose regimens used in conventional RT in primary (**a**, top) and adjuvant (**b**, center) palliative intention as well as primary SBRT (**c**, bottom), whereby for the latter only fractionations mentioned at least twice are listed; x‑axis: number of radiation oncologists indicating that they use the particular treatment regimen frequently; y‑axis: all mentioned treatment regimens, from top to bottom with increasing EQD2

There were a variety of dose regimens (*n* = 15) that were most frequently used in conventional RT (see Fig. 2a). The most commonly used dose regimen was 30 Gy in 10 fractions, with 70% (*n* = 56) of radiation oncologists applying it frequently. The following fractionation schemes were also commonly used: 20 Gy in 4 fractions (25%, *n* = 20), 35 Gy in 14 fractions (24%, *n* = 19) and 36 Gy in 12 fractions (21%, *n* = 18). Fewer than one in four of all participants frequently used schemes such as 8 Gy in a single fraction (19%, *n* = 15), 40 Gy in 20 fractions (11%, *n* = 9), 39 Gy in 13 fractions (9%, *n* = 7) and 37.5 Gy in 15 fractions (5%, *n* = 4). In total, a specific dose regimen was reported as “frequently used” 159 times, as multiple answers were allowed in this question (e.g., one participant may have reported three or four most frequently used dose regimens). In 75% (*n* = 119/159), a dose regimen was specified in which an equivalent total dose in 2 Gy (EQD2; a/ß of 10 applied for all calculations) of at least 32.5 Gy (i.e., 30 Gy in 10 fractions) is applied. A cumulative dose of 36.5 EQD2 (i.e., 35 Gy in 14 fractions) and more was specified in 39% (*n* = 62/159).

**Fig. 3:**
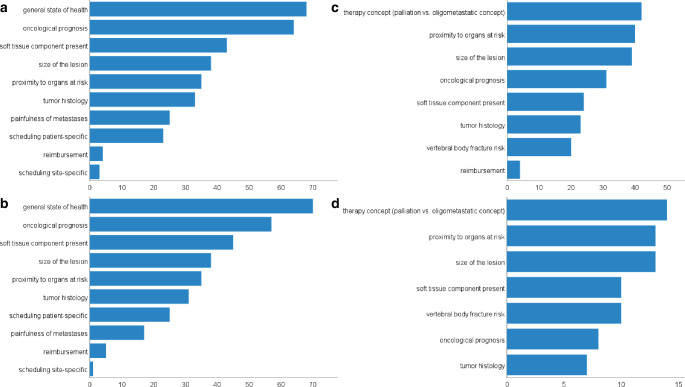
Bar charts for factors influencing the choice of dose regimens used in conventional RT in primary (3**a**, top) and adjuvant (3**b**, second from top) palliative intention as well as primary (1**c**, second from bottom) and adjuvant SBRT (1**d**, bottom); x‑axis: number of radiation oncologists considering the particular factor an important influencing factor for choice of dose regimen; y‑axis: all mentioned factors, from top to bottom in decreasing order of importance

The three most important factors for determination of the dose regimen were general state of health (85%, *n* = 68), oncological prognosis (80%, *n* = 64) and the presence of a soft tissue component of the metastasis (54%, *n* = 43). Figure 3a depicts all factors that influenced the dose regimen. Less than 50% of all participants considered the following items to be decisive for the dose regimen: Size of the lesion (48%, *n* = 38), proximity to organs at risk (44%, *n* = 35), tumor histology (41%, *n* = 33) and pain level of metastases (31%, *n* = 25). Patient-related scheduling reasons are among the most important factors for dose prescription for almost a third of all participants (29%, *n* = 23) and financial reimbursement was among the most important factors of influence for 5% (*n* = 4) of participants. Site-related scheduling (4%, *n* = 3) plays an important role in the determination of the dosage concept for individual participants of the survey.

### Section 2—conventional RT in adjuvant palliative intention

The annual number of patients treated with adjuvant palliative conventional RT was reported with 1–50 by 73% (*n* = 58), 51–100 by 18% (*n* = 14), 101–300 by 8% (*n* = 6) and 501–1000 by 3% (*n* = 2) of participants.

RT treatment planning was CT based only in 56% (*n* = 45), whereas 44% (*n* = 35) of participants used CT and MRI for treatment planning. The most used irradiation technique for conventional adjuvant palliative RT was VMAT, with 68% (*n* = 54) of radiation oncologists applying it frequently, followed by 3D-RT (48%, *n* = 38), IMRT (26%, *n* = 21) and Tomotherapy (5%, *n* = 4). In those cases where participants were aware of the type of surgical material used for stabilization (*n* = 53), it was indicated that titanium implants were used in the vast majority of cases (*n* = 50; 94%). Three participants (6%) stated that carbon fiber implants are mostly used at their institution.

Fourteen different dose regimens were identified as “most commonly applied” in conventional adjuvant palliative RT (see Fig. 2b). By far the most frequently mentioned regimen was 30 Gy in 10 fractions (64%, *n* = 51), followed by 35 Gy in 14 fractions (28%, *n* = 22). Fewer than one fourth of all participants frequently used the following schemes: 36 Gy in 12 fractions (19%, *n* = 15), 40 Gy in 20 fractions (15%, *n* = 12), 20 Gy in 4 fractions (10%, *n* = 8), 39 Gy in 13 fractions (8%, *n* = 6) and 37.5 Gy in 15 fractions (5%, *n* = 4). From a total of 128 designations of a “frequently used” dose concept for conventional adjuvant palliative RT, 91% (*n* = 116/128) of responses contained a treatment scheme with a cumulative dose of at least 32.5 Gy EQD2. A cumulative dose of at least 36.5 Gy EQD2 was reported in 50% (64/128).

The influencing factors for determining the dose regimen are fairly similar to those for primary palliative RT. As in conventional primary palliative RT, the top three factors were patient’s general state of health (88%, *n* = 70), oncological prognosis (71%, *n* = 57) and whether a soft tissue component is present (56%, *n* = 45; see Fig. 3b). For more than a third of all participants, size of the lesion (48%, *n* = 38), proximity to organs at risk (44%, *n* = 35) and tumor histology (39%, *n* = 31) were among the most relevant factors. As for conventional primary palliative RT, patient-related scheduling reasons were among the most important factors for dose prescription for almost a third of all participants (31%, *n* = 25). Pain level of metastases and financial reimbursement were among the most important factors of influence for 21% (*n* = 17) and 6% (*n* = 5) of participants, respectively. Site-related scheduling (1%, *n* = 1) played an important role in the determination of the dosage concept for one participant of the survey.

The majority of participants tended to use extended target volumes. Regarding the craniocaudal extent of the target volume, 49% (*n* = 39) of participants reported to regularly include all vertebral segments over which the surgical instrumentation extended. One quarter (*n* = 20) of participants indicated that they regularly define a smaller target volume including the vertebral segment above and below the affected vertebral segment, as well as the affected vertebral segment itself. 11% (*n* = 9) of radiation oncologists routinely opted for an even more reduced target volume including the affected vertebral body only. All vertebral bodies in the region of the instrumentation affected by metastases were systematically included in the target volume by 49% (*n* = 39). One participant specified an individual response, stating that the craniocaudal extent of the target volume depended on the radiological appearance/histology, including coverage of all vertebral segments over which the surgical instrumentation extended for lytic metastases, and one vertebral segment above and below the affected vertebral segment, as well as the affected vertebral segment itself in case of non-lytic metastases.

As with the definition of the craniocaudal extent of treatment volumes, there was no uniform approach to target volume delineation regarding the inclusion of surgical instrumentation and track. 45% (*n* = 36) of participants reported they regularly included the entire surgical instrumentation (screws and rods) outside of the bone tissue. A systematic inclusion of the entire surgical tract dorsal to the vertebral body/bodies in the target volume was performed by 24% (*n* = 19) of radiation oncologists. 43% (*n* = 34) of participants reported they included neither the surgical instrumentation outside of the bone tissue nor the entire surgical tract dorsal to the vertebral body/bodies. One participant specified that the approach in treatment volume delineation depended on the method of surgery, stating that the whole instrumentation was included, except when pedicle screws were implanted percutaneously in non-affected vertebral segments. In the latter case, the one participant stated to not include the instrumentation in the target volume.

The heterogeneous responses regarding target volume definition in conventional adjuvant palliative RT were somewhat suspected by us in advance, given the lack of specific guidelines. In this context, and given the fact that we are currently preparing a prospective trial on target volume delineation in this therapy situation at our institution, we asked whether patients could currently be enrolled in a prospective study at the institution of the survey participant. All participants replied that they had no possibility to enroll patients in a prospective trial on conventional adjuvant palliative RT at their institution.

Radiooncological follow-up of patients with spinal metastases treated with RT in a palliative intention was not consistently offered. Regular follow-up was carried out by 69% (*n* = 55) of participants. Follow-up was performed in case of new complaints in 23% (*n* = 18). The following responses were provided by individual participants only: Scheduling of follow-up visits depending on the general state of health and/or patient’s prognosis (4%, *n* = 3), no radiooncological follow-up (3%, *n* = 2), a single radiooncological follow-up visit three months after palliative RT (1%, *n* = 1) and radiooncological follow-up in cooperation or coordination with the clinic primarily responsible for the oncologic treatment of the patient (1%, *n* = 1).

### Section 3—SBRT in primary intention

Primary SBRT i.e., without prior surgical intervention, was offered at 65% (*n* = 52) of participant’s institutions. For university hospitals, this was indicated in 93% (*n* = 25), for non-university hospitals in 50% (*n* = 9) and for medical practices/ambulatory health care in 49% (*n* = 15) of responses.

SBRT for spinal metastases was much less frequently carried out than palliative RT. Of the radiation oncologists stating that primary SBRT is offered at their institution, a majority of 87% (*n* = 45) reported annual patient numbers of 1–50, 12% (*n* = 6) indicated 51–100 and 2% (*n* = 1) reported 500.

Treatment planning was CT- and MRI-based in most cases (73%, *n* = 38). However, 27% (*n* = 14) of participants stated to perform primary SBRT CT-based only. PET, if applicable, was used in addition to CT and MRI for target volume definition by three radiation oncologists (6%). Participants at institutions where SBRT for spinal metastases was performed mostly (94%, *n* = 49) reported using a linear accelerator for this purpose. Three (6%) radiation oncologists indicated to regularly use a Cyberknife (Accuray, Sunnyvale, CA, USA). Of these, two reported using a Cyberknife exclusively for SBRT of spinal metastases.

Specific dose regimens regularly used for primary SBRT were reported by 47 participants. As many as 40 regimens for dose prescription, both without and with simultaneous integrated boost (SIB), were reported, ranging from single-fraction SBRT to as many as 16 fractions (for full list of reported dose regimens, see Appendix Table 2). 30 (64%) radiation oncologists specified one regimen that they used most often. In the other cases (*n* = 17, 36%), several dose regimens were reported. 15 dose regimens were identified that were mentioned by more than one participant (see Fig. 2c). The dose regimens most frequently mentioned were 27 Gy in 3 fractions and 30 Gy in 5 fractions (*n* = 8, 17% each), 25 Gy in 5 fractions plus SIB with 40 Gy in 5 fractions and 30 Gy in 10 fractions plus SIB with 48.5 Gy in 10 fractions (*n* = 6, 13% each), 35 Gy in 5 fractions (*n* = 5, 11%), 24 Gy in 3 fractions (*n* = 4, 9%) as well as 30 Gy in 10 fractions plus SIB with 40 Gy in 10 fractions, 25 Gy in 5 fractions and 30 Gy in 6 fractions (*n* = 3, 6% each). In three cases, additional information on prescription isodoses was provided. The respective treatment concepts were: 25 Gy in 5 fractions prescribed to the 60% isodose line encompassing the planning target volume (PTV), 30 Gy in 5 fractions prescribed to the 80% isodose line and 25 Gy in 5 fractions plus SIB with 6–8 Gy single dose prescribed to the 65–80% isodose line encompassing the gross tumor volume (GTV) + 5 mm.

Among the most reported reasons for selecting the dose regimen in primary SBRT, as reported by 50 participants, were therapy goal i.e., palliation vs. ablation of oligometastasis with a curative intent (*n* = 42, 84%), proximity to organs at risk (*n* = 40, 80%) and size of the lesion (*n* = 39, 78%, see Fig. 3c). For more than half of the participants whose institution offered SBRT (*n* = 31, 62%), oncological prognosis was an important decision criterion in dose determination. Other important factors included presence of soft tissue component (*n* = 24, 48%), tumor histology (*n* = 23, 46%) and vertebral body fracture risk (*n* = 20, 40%). Reimbursement was an important factor of influence for four (8%) participants.

Fifteen out of 49 (31%) radiation oncologists who delivered primary SBRT stated they did not follow specific contouring recommendations. By far the most followed ICG (*n* = 29, 59%) were the International Spine Radiosurgery Consortium consensus guidelines for target volume definition in spinal stereotactic radiosurgery [15]. Target volume definition according to the DOSIS trial [18] was performed by 8% (*n* = 4). The following contouring recommendations for primary SBRT were mentioned by individual participants: International consensus recommendations for target volume delineation specific to sacral metastases and spinal stereotactic body radiation therapy (SBRT) [17], SABR-COMET trial [6], RTOG 0631 trial [14] and SPIN-MET trial [19] (*n* = 1 each). One participant specified to contour the lesion only plus PTV for small metastases. A tabular overview of the publications mentioned, including the respective recommendations, can be found in Table 1. ICG for adjuvant SBRT i.e., Consensus Contouring Guidelines for Post-Operative Stereotactic Body Radiation Therapy (SBRT) for Metastatic Solid Tumor Malignancies to the Spine [16] were also mentioned in this context.

**Table 1:** Tabular overview of all mentioned publications regarding target volume definition in primary and post-operative, adjuvant SBRT of spinal metastases with their respective frequency of indication (*n*) by participants of this survey and the specified recommendations

Publication	*n* =	Recommendation
International Spine Radiosurgery Consortium consensus guidelines for target volume definition in spinal stereotactic radiosurgery Cox et al., Int J Radiat Oncol Biol Phys, 2012 Aug 1 [15]	29	Delineation based on all available clinical information and imaging modalities (CT, MRI, myelography, plain film, functional imaging)
		GTV: macroscopic tumor with all eventual epidural and paraspinal components
		CTV: with bony margin enclosing abnormal bone marrow signal suspicious for micrometastases and regular bone marrow as well to account for subclinical spread; the exact extension depending on the location of the metastasis in the vertebrae (detailed recommendations available)
		PTV: with margin ≤ 3 mm with uniform 3D expansion, showing no overlap with spinal cord or cauda equina
Consensus Contouring Guidelines for Post-Operative Stereotactic Body Radiation Therapy (SBRT) for Metastatic Solid Tumor Malignancies to the Spine Redmond et al., Int J Radiat Oncol Biol Phys, 2017 Jan 1 [16]	9	Delineation on CT + MRI
		GTV: macroscopic tumor with all eventual epidural and paraspinal components
		CTV: inclusion of adjacent anatomic compartments at risk of microscopic disease; no inclusion of surgical instrumentation and tract unless at risk of tumor involvement, anatomical expansion up to 5 mm regarding eventual epidural and paraspinal components
		PTV: expansion of CTV up to 2.5 mm, possible modification regarding critical organs at risk
Fractionated radiosurgery for painful spinal metastases: DOSIS—a phase II trial Guckenberger et al., BMC Cancer, 2012 Nov 19 [18]	4	Delineation on MRI or CT
		GTV: macroscopic tumor
		PTV boost: all macroscopically involved substructures of the vertebrae
		PTV elective: entire vertebrae
International consensus recommendations for target volume delineation specific to sacral metastases and spinal stereotactic body radiation therapy (SBRT) Dunne et al., Radiother Oncol, 2020 Apr [17]	1	Delineation on CT + MRI
		CTV: entire segment containing the metastasis + immediate adjacent bony anatomic segment at risk of microscopic spread; the exact extension depending on the location of the metastasis in the vertebrae (detailed recommendations available)
Stereotactic ablative radiotherapy for comprehensive treatment of oligometastatic tumors (SABR-COMET): study protocol for a randomized phase II trial Palma et al., BMC Cancer, 2012 Jul 23 [8]	1	Delineation on CT ± MRI ± PET
		GTV: macroscopic tumor
		CTV: whole vertebrae might be delineated as CTV, as per institutional standard
RTOG 0631 phase 2/3 study of image guided stereotactic radiosurgery for localized (1–3) spine metastases: phase 2 results Ryu et al., Pract Radiat Oncol, 2014 Mar-Apr [14]	1	Delineation on CT + MRI
		PTV: vertebral body, both pedicles, all eventual epidural (if gap ≥ 3 mm between spinal cord and epidural lesion) and paraspinal components (≤ 5 cm in the greatest dimension)
SPIN-MET trial (Efficacy of Dose Intensified Radiotherapy of Spinal Metastases by Hypofractionated Radiation and IGRT hfSRT Mediated Boost) study protocol not published https://clinicaltrials.gov/show/NCT01849510	1	Information not publicly accessible

### Section 4—SBRT in adjuvant intention

A minority of 17 (21%) participants indicated that SBRT in adjuvant intention was carried out at their institution. This was reported for university hospitals by 33% (*n* = 9), for non-university hospitals by 11% (*n* = 2) and for medical practices/ambulatory health care by 17% (*n* = 17) of participants.

Reported annual patient numbers for SBRT in adjuvant intention were relatively low, with 1–50 in 76% (*n* = 13), 51–100 in 12% (*n* = 2), 101–300 in 6% (*n* = 1) and 301–500 in likewise 6% (*n* = 1).

As in primary SBRT, treatment planning was CT- and MRI-based in most cases (76%, *n* = 13). However, 24% (*n* = 4) indicated planning was solely CT-based.

Fifteen participants indicated most frequently used dose regimens, resulting in a total of 27 different regimens reported. The indicated dose regimens ranged from single-fraction SBRT to as many as 20 fractions, and as in primary SBRT, both regimens with and without SIB were reported. 9 (60%) radiation oncologists indicated one specific dose regimen they used most for adjuvant SBRT. The other six (40%) participants reported two or more regimens they frequently used. Only one dosing regimen was mentioned twice, namely 30 Gy in 10 fractions plus SIB with 48.5 Gy in 10 fractions. All other schemes, for which an overview can be found in Appendix Table 3, were reported only once. Two participants provided additional information on dose prescription i.e., 25 Gy in 5 fractions prescribed to the isodose line encompassing PTV and 30 Gy in 5 fractions prescribed to the 80% isodose line.

The factors of influence on the dose regimen are fairly similar to those for primary SBRT (see Fig. 2d). In adjuvant SBRT therapy goal i.e., palliation vs. ablation of oligometastasis with curative intent (*n* = 14, 82%), proximity to organs at risk (*n* = 13, 76%) and size of the lesion (*n* = 14, 82%) were also identified as the three most important ones. For more than half of radiation oncologists at whose institution adjuvant SBRT was performed, factors including presence of soft tissue component and vertebral body fracture risk (*n* = 10, 59%) were also among the most important factors influencing the dose regimen. Other factors mentioned were oncological prognosis (*n* = 8, 47%) and tumor histology (*n* = 7, 41%).

Thirty-five percent (*n* = 6) indicated that they did not follow any specific guidelines for target volume delineation in adjuvant SBRT. A majority of 52% (*n* = 9) of participants specified to consider the Consensus Contouring Guidelines for Post-Operative Stereotactic Body Radiation Therapy (SBRT) for Metastatic Solid Tumor Malignancies to the Spine [16] for target volume delineation. One participant reported to contour in analogy to the DOSIS trial [18] in addition to the beforementioned Consensus Contouring Guidelines. Another participant stated to use recommendations for target volume definition of the RTOG 0631 [14] trial. Both studies contain target volume contouring strategies for primary SBRT, and it remains unclear to what extent participants extrapolate these to the adjuvant setting.

## Discussion

Palliative RT of spinal metastases is a frequently performed therapy. Accordingly, participants reported considerable experience. This is particularly true for primary palliative RT, where most participants (64%) reported an annual patient volume of more than 50. There was also a considerable number of participants working in centers with a large number of patients with annual case numbers of 300 or more (*n* = 11 for primary and *n* = 6 for postoperative palliative RT), reflecting their expertise in this field.

Overall, our survey indicates that many different treatment regimens are used and that standardized guidelines are warranted. The study identified most commonly used dose regimens i.e., 30 Gy in 10 fractions, 20 Gy in 5 fractions, 35 Gy in 14 fractions and 36 Gy in 12 fractions, reported by > 20% of participants. Factors influencing the choice of dose regimen in conventional primary and adjuvant RT were nearly identical in rank order, with the top three items being patient’s general state of health, oncological prognosis, and soft tissue component present. Tumor histology was reported to only play a minor important role in determining the dosage regimen (for primary and adjuvant RT 41% and 39%, respectively). This is in line with data showing no significant improvement in local control at one year with doses beyond 30 Gy in 10 fractions for radioresistant tumors [20]. The pain level of the bone metastases also did not play an important role in dose determination (for primary and adjuvant RT 31% and 21%, respectively), in line with data showing a sufficient pain response for various dose schemes [10]. RT technique ap-pa (for primary and adjuvant RT 1% and 0%, respectively 1/0%) seems almost completely obsolete, whereas 3D-RT still plays a relevant role in conventional RT (for primary and adjuvant RT 50% and 48%, respectively).

Target volume definition in conventional adjuvant RT was heterogenous, with a tendency towards extended treatment volumes. All vertebral segments over which the surgical instrumentation extended were included by 49%, and the entire surgical instrumentation (screws and rods) outside of the bone tissue were included by 45% of participants. However, 43% included neither the surgical instrumentation outside of the bone tissue nor the entire surgical tract dorsal to the vertebral body/bodies, but only 11% routinely including the affected vertebral body only. There are no guidelines for target volume definition in conventional adjuvant RT, and the heterogeneity in responses regarding strategies in target volume delineation were therefore to some extent expectable. To provide a solid data basis for contouring recommendations in this situation, patterns-of-failure analyses are required. One prerequisite for such analyses would be systematic radiation oncology follow-up visits, as offered by 69% of participants. Prospective studies would also be very desirable to provide further evidence regarding target volume definition. A prospective study in this regard is currently not performed at any institution of the participants of this survey. However, we are currently preparing a prospective trial on conventional adjuvant RT comparing larger with reduced treatment volumes with regard to local tumor control.

The survey showed that a majority of institutions (65%) offers SBRT for spinal metastases. However, patients do not have access to SBRT in every institution, and it is especially limited in the adjuvant setting, where only 21% of participants report to perform it at their institution. The lack of widespread availability of SBRT in the adjuvant setting may impose further consequences in individual cases. It is possible that in the absence of adjuvant SBRT, conventional RT may be utilized, or that it might also needs to be considered regarding the surgical approach (decompression and stabilization vs. favor of en-bloc resection, when adjuvant SBRT is not available).

In primary SBRT, there is a great variability of dose concepts (for primary and adjuvant SBRT *n* = 40 and 27, respectively) that were utilized, with 15 regimens being applied by two or more participants. The most frequently mentioned regimens were 30 Gy in 5 fractions, 27 Gy in 3 fractions, 25 Gy in 5 fractions plus SIB with 40 Gy in 5 fractions, 30 Gy in 10 fractions plus SIB with 48.5 Gy in 10 fractions and 35 Gy in 5 fractions. While SIB concepts are being administered in palliative RT by a small number of participants, they have a greater relevance in daily practice of SBRT. It is unclear as to why some of the regimens applied both in the primary and adjuvant setting were reported using a stereotactic approach e.g., 12 Gy in 3 fractions, 20 Gy in 5 fractions, 30 Gy in 10 fractions or 37.5 Gy in 15 fractions. However, this manuscript presents all responses as received. The factors influencing the choice of dose regimen were clearly different in SBRT compared to conventional RT, with top three items being therapy goal (palliation vs. ablation of oligometastasis with a curative intent), proximity to organs at risk and size of the lesion. In contrast to conventional RT, time and, secondarily, reimbursement did not play a relevant role. The finding that therapy goal was the most important influencing factor for dose prescription in SBRT might seem surprising, as SBRT is known to be effective both as ablative therapy [8] and for pain control in a palliative setting [5]. Whether the information reflects that SBRT was predominantly performed in the context of oligometastatic treatment concepts can only be hypothesized.

Fifty-nine percent of participants followed ICG for target volume delineation in primary and adjuvant SBRT. Few participants defined target volumes in accordance with proposed strategies of prospective studies. Some of the recommendations differ substantially, and as in adjuvant conventional RT, patterns-of-failure analyses and prospective data are desirable for valid, consistent target volume delineation strategies. A recent analysis strongly supports the systematic application of ICG in spine SBRT as it showed that deviation from ICG is significantly associated with inferior local tumor control [21].

This study has limitations due to its design as an anonymous online survey. There is no control on whether a participant has completed the survey more than once. Thus, it cannot be excluded with certainty that therapy strategies of individual participants are overrepresented in the results. The survey was conducted at the individual level, not at the department level. While the number of participants is representative at *n* = 80, it is unknown how many different facilities are represented by the survey. The survey depicts a comprehensive representation of the RT practice in spinal metastases of DEGRO members, however, it may not represent an overview of overall radiotherapy practice in Germany, as some centers may be overrepresented, or the geographical distribution of participants may be uneven. Nevertheless, the personal information requested in the survey showed that 21 chief physicians participated, meaning that the survey represents at least 21 different facilities, very likely more.

## Conclusion

We provide the first representative depiction of the radiation oncology treatment patterns of care of spinal metastases both with regard to conventional palliative RT as well as SBRT among DEGRO members.

For conventional RT, answers regarding dose regimen showed congruence to some extent, with 30 Gy in 10 fractions being the dose regimen regularly used by a majority of participants both in primary and adjuvant RT. However, many different dose regimens are regularly used by DEGRO members, and target volume definition is heterogeneous. As spinal metastases are a common RT indication, further research such as analyses of failure patterns and especially prospective studies are needed to provide a solid data basis for detailed recommendations on conventional RT.

Primary SBRT is accessible at a majority of institutions (65%), however, adjuvant SBRT is not comprehensively offered. This might impact treatment regimen in specific cases. ICG for target volume delineation exist but are not always applied. Patterns-of-failure analyses and prospective data are desirable for valid, consistent target volume delineation strategies.

## Appendix

**Appendix Questionnaire**: Original questionnaire in German language (above) and a translated version in English language (below).

### ORIGINAL QUESTIONNAIRE IN GERMAN

#### Titel: Therapiekonzepte in der Bestrahlung von Wirbelsäulenmetastasen

Teil 1 – Konventionelle Bestrahlung von Wirbelsäulenmetastasen in primärer palliativer Intention ohne vorausgegangene Operation (keine SBRT)

Bitte beantworten Sie die folgenden Fragen ausschließlich bezogen auf die primäre Bestrahlung von Wirbelsäulenmetastasen in palliativer Intention ohne vorausgegangene Operation. Bitte machen Sie hier nur Angaben zur konventionellen Bestrahlung (keine SBRT).

1. Wie viele Patient*innen mit Wirbelsäulenmetastasen werden an Ihrem Standort jährlich ungefähr in primärer palliativer Intention behandelt?
  *Geben Sie eine Zahl ein [Freitext]*
2. Welches bzw. welche der folgenden Dosisschemata verwenden Sie bei der primären palliativen Bestrahlung von Wirbelsäulenmetastasen am häufigsten?
  *Wählen Sie eine oder mehr Antworten: 8* *Gy GD, 8* *Gy ED; 20* *Gy GD, 5* *Gy ED; 30* *Gy GD, 3* *Gy ED; 35* *Gy GD, 2,5* *Gy ED; 36* *Gy GD, 3* *Gy ED; 39* *Gy GD, 3* *Gy ED; 40* *Gy GD, 2* *Gy ED; 42* *Gy GD, 3* *Gy ED; andere [Freitext]*
3. Was sind für Sie hierbei die wichtigsten Faktoren zur Festlegung des Dosiskonzepts?
  *Wählen Sie eine oder mehr Antworten: Größe der Läsion; Tumorhistologie; vorhandene Weichteilkomponente; Lagebeziehung zu Risikostrukturen; Allgemeinzustand des Patienten/der Patientin; Schmerzhaftigkeit der Metastasen; Onkologische Prognose des Patienten/der Patientin; Vergütung; Terminierung Standortbezogen (z.* *B. Geräteauslastung); Terminierung Patientenbezogen (z.* *B. geplante Systemtherapie); andere [Freitext]*
4. Auf Basis welcher Bildgebung erfolgt die Bestrahlungsplanung hierbei regelhaft?
  *Wählen Sie eine Antwort: CT; CT und MRT; andere [Freitext]*
5. Welche Bestrahlungstechnik wird hierbei am häufigsten verwendet?
  *Wählen Sie eine oder mehr Antworten: ap-pa; 3D; VMAT; IMRT; Tomotherapie-Technik; andere [Freitext]*

Te﻿il 2 – Konventionelle Bestrahlung von Wirbelsäulenmetastasen in adjuvanter palliativer Intention nach vorausgegangener Operation (keine SBRT)

Bitte beantworten Sie die folgenden Fragen ausschließlich bezogen auf die adjuvante Bestrahlung von Wirbelsäulenmetastasen in palliativer Intention nach vorausgegangener Operation. Bitte machen Sie hier nur Angaben zur konventionellen Bestrahlung (keine SBRT).

6. Wie viele Patient*innen mit Wirbelsäulenmetastasen werden an Ihrem Standort jährlich ungefähr in adjuvanter palliativer Intention behandelt?
  *Geben Sie eine Zahl ein [Freitext]*
7. Welches Operationsmaterial wird an Ihrem Standort bei der dorsalen Stabilisierung am häufigsten verwendet?
  *Wählen Sie eine Antwort: Carbon; Titan; nicht bekannt*
8. Welches bzw. welche der folgenden Dosisschemata verwenden Sie bei der adjuvanten palliativen Bestrahlung von Wirbelsäulenmetastasen am häufigsten?
  *Wählen Sie eine oder mehr Antworten: 8* *Gy GD, 8* *Gy ED; 20* *Gy GD, 5* *Gy ED; 30* *Gy GD, 3* *Gy ED; 35* *Gy GD, 2,5* *Gy ED; 36* *Gy GD, 3* *Gy ED; 39* *Gy GD, 3* *Gy ED; 40* *Gy GD, 2* *Gy ED; 42* *Gy GD, 3* *Gy ED; andere [Freitext]*
9. Was sind für Sie hierbei die wichtigsten Faktoren zur Festlegung des Dosiskonzepts?
  *Wählen Sie eine oder mehr Antworten: Größe der Läsion; Tumorhistologie; vorhandene Weichteilkomponente; Lagebeziehung zu Risikostrukturen; Allgemeinzustand des Patienten/der Patientin; Schmerzhaftigkeit der Metastasen; Onkologische Prognose des Patienten/der Patientin; Vergütung; Terminierung Standortbezogen (z.* *B. Geräteauslastung); Terminierung Patientenbezogen (z.* *B. geplante Systemtherapie); andere [Freitext]*
10. Auf Basis welcher Bildgebung erfolgt die Bestrahlungsplanung hierbei regelhaft?
  *Wählen Sie eine Antwort: CT; CT und MRT; andere [Freitext]*
11. Welche Bestrahlungstechnik wird hierbei am häufigsten verwendet?
  *Wählen Sie eine oder mehr Antworten: ap-pa; 3D; VMAT; IMRT; Tomotherapie-Technik; andere [Freitext]*
12. Welche der folgenden Aussagen treffen auf das regelhaft von Ihnen gewählte Vorgehen bei der Zielvolumenkonturierung (CTV) in der konventionellen Bestrahlung in adjuvanter palliativer Intention für Patient*innen nach dorsaler Stabilisierung zu?
  Teil 1: Einschluss von Wirbelkörperhöhen
  *Wählen Sie eine oder mehr Antworten. „Betroffene Wirbelkörperhöhe“ meint hier jene Wirbelkörperhöhe, aufgrund derer die dorsale Stabilisierung erfolgte: Einschluss aller Wirbelkörperhöhen, über welche sich die Instrumentierung erstreckt; Einschluss aller Wirbelköperhöhen im Bereich der Instrumentierung, welche metastatisch befallen sind; Einschluss einer Wirbelkörperhöhe über- und unterhalb der betroffenen Höhe sowie die betroffene Wirbelkörperhöhe selbst; Einschluss lediglich der betroffenen Wirbelkörperhöhe; andere [Freitext]*
13. Teil 2: Operationsmaterial und Zugangsweg
  *Wählen Sie eine oder mehr Antworten: Einschluss der gesamten Instrumentierung (Schrauben und Stäbe außerhalb des Knochengewebes); Einschluss des gesamten chirurgischen Zugangswegs dorsal der Wirbelkörper; Weder Einschluss von Operationsmaterial außerhalb des Knochengewebes noch Einschluss des chirurgischen Zugangswegs; andere [Freitext]*
14. Besteht an Ihrem Standort die Möglichkeit, Patient*innen mit Bestrahlung von Wirbelsäulenmetastasen in adjuvanter palliativer Intention in eine diesbezügliche prospektive Studie einzuschließen?
  *Wählen Sie eine Antwort: nein; ja, in die folgende Studie(n) [Freitext]*
15. Welche Aussage zur klinischen radioonkologischen Nachsorge nach Bestrahlung von Wirbelsäulenmetastasen in palliativer Intention (primär und adjuvant) trifft für Ihren Standort zu?
  *Wählen Sie eine Antwort: Eine klinische radioonkologische Nachsorge erfolgt nicht.; Eine klinische radioonkologische Kontrolle erfolgt nur im Fall neuer Beschwerden.; Es erfolgt eine regelmäßige radioonkologische klinische Nachsorge.; andere [Freitext]*
16. Welche Aussage zur bildgebenden radioonkologischen Nachsorge nach Bestrahlung von Wirbelsäulenmetastasen in palliativer Intention (primär und adjuvant) trifft für Ihren Standort zu?
  *Wählen Sie eine Antwort. Die Bildgebung bezieht sich hierbei dediziert auf die behandelte Knochenmetastase(n): Eine dedizierte bildgebende Nachsorge erfolgt nicht.; Eine dedizierte bildgebende Nachsorge wird nur im Fall neuer Beschwerden veranlasst.; Eine dedizierte bildgebende Nachsorge wird durchgeführt, um die lokale Kontrolle nach Bestrahlung zu beurteilen.; andere [Freitext]*

Teil 3 – Primäre stereotaktische Bestrahlung (SBRT) von Wirbelsäulenmetastasen ohne vorausgegangene Operation

Bitte beantworten Sie die folgenden Fragen ausschließlich bezogen auf die primäre stereotaktische Bestrahlung von Wirbelsäulenmetastasen. Fragen zur adjuvanten SBRT folgen im Anschluss gesondert.

Die folgenden Fragen beziehen sich sowohl auf die Bestrahlung in palliativer Intention als auch im Rahmen von Oligometastasierungskonzepten.

17. Wird an Ihrem Zentrum eine stereotaktische Bestrahlung von Wirbelsäulenmetastasen angeboten?
  *Wählen Sie eine Antwort: nein; ja*
18. Wie viele Patient*innen mit Wirbelsäulenmetastasen werden an Ihrem Standort jährlich ungefähr in primärer Intention mit SBRT behandelt?
  *Geben Sie eine Zahl ein [Freitext]*
19. Welches oder welche Dosisschemata verwenden Sie bei der primären SBRT von Wirbelsäulenmetastasen am häufigsten?
  *Schreiben Sie einen kurzen Text [Freitext]*
20. Was sind für Sie hierbei die wichtigsten Faktoren zur Festlegung des Dosiskonzepts?
  *Wählen Sie eine oder mehr Antworten: Größe der Läsion; Tumorhistologie; vorhandene Weichteilkomponente; Lagebeziehung zu Risikostrukturen; Frakturrisiko des Wirbelkörpers; Therapiekonzept (Palliation vs. Oligometastasierungskonzept); Prognose des Patienten/der Patientin; Vergütung; andere [Freitext]*
21. Auf Basis welcher Bildgebung erfolgt die Bestrahlungsplanung hierbei regelhaft?
  *Wählen Sie eine Antwort: CT; CT und MRT; andere [Freitext]*
22. Berücksichtigen Sie hierbei regelhaft bestimmte Konturierungsempfehlungen?
  *Wählen Sie eine oder mehr Antworten: nein; ja: International Spine Radiosurgery Consortium consensus guidelines for target volume definition in spinal stereotactic radiosurgery** (Cox et al. *[2012]*); andere [Freitext]*

Teil 4 – Adjuvante stereotaktische Bestrahlung (SBRT) von Wirbelsäulenmetastasen mit vorausgegangener Operation

Bitte beantworten Sie die folgenden Fragen ausschließlich bezogen auf die postoperative adjuvante stereotaktische Bestrahlung von Wirbelkörpermetastasen, sofern nicht anders ausgewiesen.

Die folgenden Fragen beziehen sich sowohl auf die Bestrahlung in palliativer Intention als auch im Rahmen von Oligometastasierungskonzepten.

23. Wie viele Patient*innen mit Wirbelsäulenmetastasen werden an Ihrem Standort jährlich ungefähr in adjuvanter Intention mit SBRT behandelt?
  *Geben Sie eine Zahl ein [Freitext]*
24. Welches oder welche Dosisschemata verwenden Sie bei der primären SBRT von Wirbelsäulenmetastasen am häufigsten?
  *Schreiben Sie einen kurzen Text [Freitext]*
25. Was sind für Sie hierbei die wichtigsten Faktoren zur Festlegung des Dosiskonzepts?
  *Wählen Sie eine oder mehr Antworten: Größe der Läsion; Tumorhistologie; vorhandene Weichteilkomponente; Lagebeziehung zu Risikostrukturen; Frakturrisiko des Wirbelkörpers; Therapiekonzept (Palliation vs. Oligometastasierungskonzept); Prognose des Patienten/der Patientin; Vergütung; andere [Freitext]*
26. Auf Basis welcher Bildgebung erfolgt die Bestrahlungsplanung hierbei regelhaft?
  *Wählen Sie eine Antwort: CT; CT und MRT; andere [Freitext]*
27. Berücksichtigen Sie hierbei regelhaft bestimmte Konturierungsempfehlungen?
  *Wählen Sie eine oder mehr Antworten: nein; ja: Consensus Contouring Guidelines for Postoperative Stereotactic Body Radiation Therapy for Metastatic Solid Tumor Malignancies to the Spine** (Redmond et al. *[2017]*); andere [Freitext]*
28. Welches Bestrahlungsgerät wird zur stereotaktischen Bestrahlung von Wirbelsäulenmetastasen (primär und adjuvant) an Ihrem Standort am häufigsten verwendet?
  *Wählen Sie eine Antwort: Linearbeschleuniger; Cyberknife*
  Wird an Ihrem Standort zur Bestrahlung von Wirbelsäulenmetastasen ausschließlich ein Cyberknife eingesetzt?
  *Wählen Sie eine Antwort: ja; nein*

Fragen zum Teilnehmenden an der Umfrage

29. Über welche Berufserfahrung in der RadioOnkologie verfügen Sie?
  *Wählen Sie eine Antwort: Assistenzarzt/-ärztin (1–3 Jahre Berufserfahrung) RadioOnkologie; Assistenzarzt/-ärztin (4–5 Jahre Berufserfahrung) RadioOnkologie; Facharzt/-ärztin RadioOnkologie; Facharzt/-ärztin Neurochirurgie; Oberarzt/-ärztin RadioOnkologie; Chefarzt/-ärztin/Leitung einer Einrichtung RadioOnkologie; andere [Freitext]*
30. In welcher Einrichtung sind Sie tätig?
  *Wählen Sie eine Antwort: Universitätsklinik; Nicht-universitäre Klinik; MVZ /Praxis; andere [Freitext]*
31. In welchem Land sind Sie tätig?
  *Wählen Sie eine Antwort: Deutschland; Österreich; Schweiz; andere [Freitext]*

### TRANSLATION OF THE QUESTIONNAIRE IN ENGLISH LANGUAGE

#### Title: Radiation concepts of spinal metastases

Part 1—Conventional radiotherapy of spinal metastases in primary palliative intention without previous surgery (no SBRT)

Please answer the following questions exclusively related to primary irradiation of spinal metastases in palliative intention without previous surgery. Please only provide information on conventional radiotherapy (no SBRT).

1. Approximately how many patients with spinal metastases are treated with primary palliative intention at your institution annually?
  *Enter a number [free text]*
2. Which dose regimen(s) do you most commonly use in primary palliative radiotherapy for spinal metastases?
  *Select one or more answers: 8* *Gy total dose (TD), 8* *Gy single dose (SD); 20* *Gy TD, 5* *Gy SD; 30* *Gy TD, 3* *Gy SD; 35* *Gy TD, 2.5* *Gy SD; 36* *Gy TD, 3* *Gy SD; 39* *Gy TD, 3* *Gy SD; 40* *Gy TD, 2* *Gy SD; 42* *Gy TD, 3* *Gy SD; other [free text]*
3. What are the most critical factors driving your decision regarding prescription dose here?
  *Select one or more answers: Size of the lesion; tumor histology; soft tissue component present; proximity to organs ot risk; patient’s general state of health; painfulness of metastases; patient’s oncological prognosis; reimbursement; scheduling site-specific (e.g., available therapy slots); scheduling patient-specific (e.g., planned systemic therapy); other [free text]*
4. Based on which imaging modality is the treatment planning usually performed here?
  *Select one answer: CT; CT and MRI; other [free text]*
5. Which irradiation technique is mainly used here?
  *Select one or more answers: ap-pa; 3D; VMAT; IMRT; Tomotherapy; other [free text]*

Part 2—Conventional radiotherapy of spinal metastases in adjuvant palliative intention after previous surgery (no SBRT)

Please answer the following questions exclusively related to adjuvant radiotherapy of spinal metastases in palliative intention after previous surgery. Please only provide information on conventional radiotherapy (no SBRT).

6. Approximately how many patients with spinal metastases are treated with adjuvant palliative intention at your institution annually?
  *Enter a number [free text]*
7. Which surgical material is mainly used for dorsal spine stabilization?
  *Select one answer: Carbon; Titanium; not known*
8. Which dose regimen(s) do you most commonly use in adjuvant palliative radiotherapy for spinal metastases?
  *Select one or more answers: 8* *Gy TD, 8* *Gy SD; 20* *Gy TD, 5* *Gy SD; 30* *Gy TD, 3* *Gy SD; 35* *Gy TD, 2.5* *Gy SD; 36* *Gy TD, 3* *Gy SD; 39* *Gy TD, 3* *Gy SD; 40* *Gy TD, 2* *Gy SD; 42* *Gy TD, 3* *Gy SD; other [free text]*
9. What are the most critical factors driving your decision regarding prescription dose here?
  *Select one or more answers: Size of the lesion; tumor histology; soft tissue component present; proximity to organs ot risk; patient’s general state of health; painfulness of metastases; patient’s oncological prognosis; reimbursement; scheduling site-specific (e.g., available therapy slots); scheduling patient-specific (e.g., planned systemic therapy); other [free text]*
10. Based on which imaging modality is the treatment planning usually performed here?
  *Select one answer: CT; CT and MRI; other [free text]*
11. Which irradiation technique is mainly used here?
  *Select one or more answers: ap-pa; 3D; VMAT; IMRT; Tomotherapy; other [free text]*
12. Which of the following statements apply to your typical approach to target volume delineation of the clinical target volume (CTV) in conventional radiotherapy in adjuvant palliative intention for patients after dorsal spine stabilization?
  Part 1: Inclusion of vertebral bodies
  *Select one or more answers. “affected vertebral body” refers to the vertebral body because of which the dorsal stabilisation was performed: Inclusion of all vertebral bodies over which the surgical instrumentation extends; inclusion of all vertebral bodies in the region of the instrumentation which are affected by metastases; inclusion of a vertebral body above and below the affected vertebral body, as well as the affected vertebral body itself; inclusion of the affected vertebral body only; other [free text]*
13. Part 2: Surgical instrumentation and surgical track
  *Select one or more answers: Inclusion of the entire surgical instrumentation (screws and rods outside of the bone tissue); Inclusion of the entire surgical tract dorsal to the vertebral body/bodies; Neither inclusion of the entire surgical instrumentation outside of the bone tissue nor the entire surgical tract dorsal to the vertebral body/bodies; other [free text]*
14. Do you have the possibility to include patients with radiation of spinal metastases in adjuvant palliative intention in a specific prospective clinical trial at your institution?
  *Select one answer: no; yes, into the following study/studies [free text]*
15. Which statement regarding clinical radio-oncological follow-up after irradiation of spinal metastases in palliative intention (primary and adjuvant) is applicable for your institution?
  *Select one answer: Clinical radio-oncological follow-up is not provided.; Clinical radio-oncological follow-up is initiated only in case of new symptoms.; Clinical radio-oncological follow-up is provided regularly.; other [free text]*
16. Which statement regarding imaging radio-oncological follow-up after irradiation of spinal metastases in palliative intention (primary and adjuvant) is applicable for your institution?
  *Select one answer. Imaging refers to the treated bone metastasis/es: Dedicated imaging follow-up is not performed.; Dedicated imaging follow-up is initiated only in case of new symptoms.; Dedicated imaging follow-up is performed to assess local control after irradiation.; other [free text]*

Part 3—Primary stereotactic radiotherapy (SBRT) of spinal metastases without previous surgery

Please answer the following questions exclusively regarding primary stereotactic radiotherapy of spinal metastases. Questions regarding adjuvant SBRT will follow in a separate section.

The following questions refer to primary SBRT in palliative intention and the context of oligometastatic disease.

17. Is stereotactic radiotherapy (SBRT) for spinal metastases offered at your center?
  *Select one answer: no; yes*
18. Approximately how many patients with spinal metastases are treated with primary SBRT at your institution annually?
  *Enter a number [free text]*
19. Which dose regimen(s) do you most commonly use in primary SBRT for spinal metastases?
  *Write a short text [free text]*
20. What are the most critical factors driving your decision regarding prescription dose here?
  *Select one or more answers: Size of the lesion; tumor histology; soft tissue component present; proximity to organs ot risk; risk of fracture of the vertebral body; therapy concept (palliation vs. oligometastatic concept); patient’s prognosis; reimbursement; other [free text]*
21. Based on which imaging modality is the treatment planning usually performed here?
  *Select one answer: CT; CT and MRI; other [free text]*
22. Do you regularly consider certain contouring recommendations for primary SBRT for spinal metastases?
  *Select one or more answers: no; yes: International Spine Radiosurgery Consortium consensus guidelines for target volume definition in spinal stereotactic radiosurgery** (Cox et al. *[2012]*); other [free text]*

Part 4—Adjuvant stereotactic radiotherapy (SBRT) of spinal metastases with previous surgery

Unless otherwise indicated, please answer the following questions exclusively related to postoperative adjuvant stereotactic irradiation of vertebral metastases.

The following questions refer to primary SBRT in palliative intention and the context of oligometastatic disease.

23. Approximately how many patients with spinal metastases are treated with adjuvant SBRT at your institution annually?
  *Enter a number [free text]*
24. Which dose regimen(s) do you most commonly use in adjuvant SBRT for spinal metastases?
  *Write a short text [free text]*
25. What are the most critical factors driving your decision regarding prescription dose here?
  *Select one or more answers: Size of the lesion; tumor histology; soft tissue component present; proximity to organs ot risk; risk of fracture of the vertebral body; therapy concept (palliation vs. oligometastatic concept); patient’s prognosis; reimbursement; other [free text]*
26. Based on which imaging modality is the treatment planning usually performed here?
  *Select one answer: CT; CT and MRI; other [free text]*
27. Do you regularly consider certain contouring recommendations for adjuvant SBRT for spinal metastases?
  *Select one or more answers: no; yes: Consensus Contouring Guidelines for Postoperative Stereotactic Body Radiation Therapy for Metastatic Solid Tumor Malignancies to the Spine** (Redmond et al. *[2017]*); other [free text]*
28. Which radiation device is mainly used for SBRT of spinal metastases (primary and adjuvant) at your institution?
  *Select one answer: Linear accelerator; Cyberknife*
  Is a Cyberknife used exclusively at your institution for the irradiation of spinal metastases?
  *Select one answer: yes; no*

Questions on the participant of the survey

29. What degree of experience in radiation oncology do you have?
  *Select one answer: Resident (1–3 years of working experience) in radiation oncology; Resident (4–5 years of working experience) in radiation oncology; specialist in radiation oncology; specialist in neurosurgery; senior physician in radiation oncology; chief physician in radiation oncology; other [free text]*
30. In which institution do you work?
  *Select one answer: University hospital; non-university hospital; ambulatory health care; other [free text]*
31. In which country do you work?
  *Select one answer: Germany; Austria; Switzerland; other [free text]*

**Table 2:** Tabular overview of all mentioned dose concepts in primary SBRT of spinal metastases and their respective frequency of indication (*n*), sorted by number of fractions and single dose per fraction in ascending order

Dose regimen	*n* =
1 × 8 Gy	1
1 × 19 Gy	1
1 × 20 Gy	1
1 × 21 Gy	1
1 × 24 Gy	1
3 × 4 Gy	1
3 × 7 Gy	1
3 × 8 Gy	4
3 × 9 Gy	8
3 × 10 Gy	1
4 × 5 Gy	2
4 × 6 Gy	1
5 × 4/7 Gy	2
5 × 4.5 Gy	2
5 × 5 Gy	3
5 × 5/6 Gy	1
5 × 5/7 Gy	1
5 × 5/8 Gy	6
5 × 6 Gy	8
5 × 7 Gy	5
5 × 8 Gy	2
6 × 5 Gy	3
6 × 6 Gy	2
6 × 7 Gy	1
6 × 8 Gy	1
6 × 9 Gy	1
6 × 10 Gy	1
7 × 5 Gy	1
8 × 4 Gy	1
8 × 5 Gy	1
10 × 3 Gy	1
10 × 3/4 Gy	3
10 × 3/4.5 Gy	1
10 × 3/4.85 Gy	6
10 × 4 Gy	2
10 × 5 Gy	1
12 × 4 Gy	1
12 × 5 Gy	1
15 × 2.5 Gy	1
16 × 2.5/3 Gy	1

**Table 3:** Tabular overview of all mentioned dose concepts in adjuvant SBRT of spinal metastases and their respective frequency of indication (*n*), sorted by number of fractions and single dose per fraction in ascending order

Dose regimen	*n* =
1 × 20 Gy	1
3 × 4 Gy	1
3 × 7 Gy	1
3 × 8 Gy	1
4 × 5 Gy	1
5 × 3/6 Gy	1
5 × 3/8 Gy	1
5 × 3.5 Gy	1
5 × 4 Gy	1
5 × 4/6 Gy	1
5 × 4/8 Gy	1
5 × 4.5 Gy	1
5 × 5 Gy	1
5 × 5/6 Gy	1
5 × 5/7 Gy	1
5 × 5/8 Gy	1
5 × 6 Gy	1
5 × 7 Gy	1
6 × 5 Gy	1
8 × 4 Gy	1
10 × 3 Gy	1
10 × 3/4 Gy	1
10 × 3/10 × 4.85 Gy	2
10 × 4 Gy	1
15 × 2.5 Gy	1
20 × 2/3 Gy	1
20 × 2.5 Gy	1
